# Meridian-Dependent Geometric Planning for Pars Plana Scleral Fixation: An Orientation-Adjusted Analytical Model

**DOI:** 10.3390/diagnostics16132122

**Published:** 2026-07-07

**Authors:** Goran Marić, Danny A. Mammo, Damir Godec, Milan Pešić, Zoran Vatavuk

**Affiliations:** 1 Department of Ophthalmology, University Hospital Center Sestre Milosrdnice, Vinogradska Cesta 29, 10000 Zagreb, Croatia; 2 Cole Eye Institute, Cleveland Clinic, Cleveland, OH 44195, USA; 3 Faculty of Mechanical Engineering and Naval Architecture, University of Zagreb, 10000 Zagreb, Croatia; 4 Barraquer Ophthalmology Centre, 08021 Barcelona, Spain

**Keywords:** scleral fixation, secondary intraocular lens, limbal ellipticity, ocular biometry, geometric planning, image-guided surgery, artificial capsular support, multi-anchor fixation

## Abstract

To establish a geometric framework for scleral fixation planning in secondary intraocular lens (IOL) implantation that accounts for corneal ellipticity and meridional orientation, and to analyze the limitations of fixed limbus-distance marking. The limbal boundary was modeled as an ellipse defined by the horizontal and vertical white-to-white (WTW) diameters. A target circumferential scleral locus is defined concentrically relative to an anatomical reference center. Fixation points are determined as a function of the meridional orientation θ using an explicit radial model of the elliptical limbus and an orientation-dependent limbal offset. The framework is analyzed for two-, three-, and four-point fixation configurations, including the effect of uniform circumferential displacement. A constant limbus-distance strategy implicitly assumes rotational invariance of limbal geometry. When the cornea is elliptical, this assumption produces predictable meridional deviations from the intended circumferential scleral locus for vertical and oblique fixation. An orientation-dependent limbal offset preserves geometric symmetry across all meridians and maintains fixation points on a consistent target locus, independent of angular configuration. Under representative biometric conditions, the maximal geometric deviation from the intended scleral locus approached approximately 0.58 mm when a constant limbus-distance strategy was applied. Scleral fixation planning can be formalized as a geometric problem governed by limbal ellipticity and meridional orientation. An explicit orientation-dependent model provides a technique-independent basis for reproducible fixation planning and reduces reliance on fixed-distance heuristics.

## 1. Introduction

Secondary intraocular lens (IOL) implantation with scleral fixation is widely used in eyes lacking adequate capsular support. Over the past decade, fixation techniques have evolved substantially, including suture-based and sutureless approaches, as well as two-, three-, and four-point configurations. Despite these technical refinements, the geometric planning of fixation points frequently remains based on fixed distances from the limbus, often derived from a single horizontal white-to-white (WTW) measurement.

Accurate prediction of effective lens position (ELP) remains one of the principal determinants of refractive outcome in both primary and secondary IOL implantation. Even small anterior–posterior displacement or subtle lens tilt may translate into measurable refractive error or higher-order optical aberrations [1,2]. In scleral fixation, the final IOL position is directly governed by the spatial configuration of fixation points; therefore, geometric planning is intrinsically linked to refractive predictability.

In routine practice, fixation distances are commonly selected using standardized limbus offsets (e.g., 2.5–3.5 mm posterior to the limbus), frequently applied uniformly across all meridians. This approach implicitly assumes that the limbal boundary is circular and rotationally invariant. However, biometric studies using anterior segment optical coherence tomography (AS-OCT) and Scheimpflug imaging have consistently demonstrated that the human cornea is characteristically elliptical, with the horizontal diameter typically exceeding the vertical diameter and exhibiting measurable interindividual variability [3,4].

**Figure 1:**
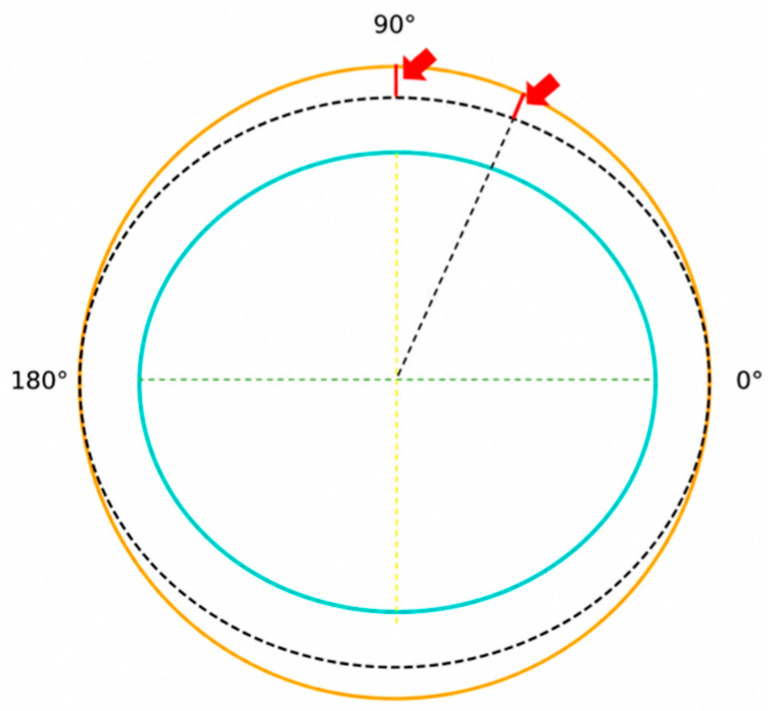
Schematic illustration of the geometric mismatch introduced by applying a constant limbus-distance marking strategy to an elliptical limbus. The limbal boundary (turquoise) is defined by horizontal (green) and vertical (yellow) WTW diameters. The dashed black circle represents a conventional constant distance circumference derived from the limbus, whereas the outer orange circle represents the intended circumferential scleral locus defined relative to the corneal center. Orientation-dependent discrepancies arise in the oblique and vertical meridians (red arrows), where the two geometries no longer coincide.

When a circular approximation is heuristically applied to define fixation distances across all meridians, systematic geometric deviations arise in vertical and oblique orientations (Figure 1). These deviations are deterministic consequences of geometric mismatch rather than manifestations of surgical variability. Because fixation geometry influences centration, tilt, and effective lens position, a planning model that incorporates the true corneal shape is warranted [5,6,7,8].

The purpose of this study is to formalize scleral fixation planning as a geometric problem governed by limbal ellipticity and meridional orientation within a pars plana surgical context familiar to vitreoretinal practice.

## 2. Materials and Methods (Geometric Basis of Scleral Fixation Planning)

### 2.1. Anatomical Reference and Pars Plana Context

Most contemporary scleral fixation techniques operate through the pars plana, minimizing anterior segment disruption [9,10]. Regardless of whether fixation is suture-based, flanged, two-point, three-point, or four-point, a shared requirement exists: fixation points must lie on a symmetric circumferential locus relative to the corneal center.

The present framework does not prescribe a specific mechanical technique. It defines fixation geometry independently of implementation, focusing exclusively on the spatial relationships between the limbus, the reference center, and the intended scleral locus.

### 2.2. Elliptical Representation of the Limbus

Let the limbus be modeled as an ellipse centered at an anatomical reference center:

- semi-major axis: a=WTW horizontal/2;
- semi-minor axis: b=WTW vertical/2.

When a≠b, the limbal radius depends on the meridional orientation (3, 11).

### 2.3. Orientation-Dependent Radial Geometry

The radial distance from the reference center to the limbus along meridian *θ* is:

$$r(\theta )=\frac{ab}{\sqrt{(b\cos\theta {)}^{2}+(a\sin\theta {)}^{2}}}$$

**Figure 2:**
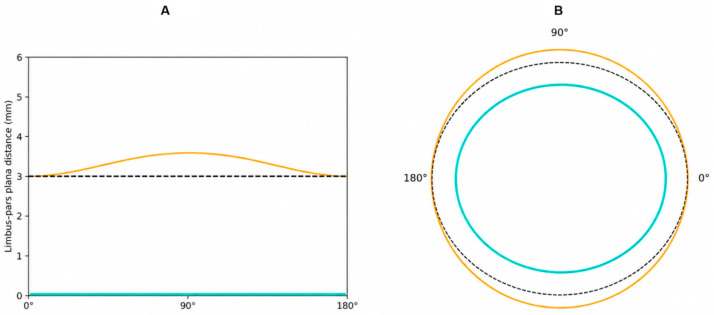
Orientation-dependent limbus–pars plana distance derived from an elliptical limbal model (horizontal WTW 11.8 mm; vertical WTW 10.63 mm). (**A**) Cartesian plot of the calculated distance versus meridional orientation. (**B**) Polar representation of the same geometry illustrating the mismatch between elliptical and circular approximations.

This expression makes explicit that the limbal radius varies continuously with orientation (Figure 2). A circular model corresponds to a = b, in which case r(θ) is constant.

## 3. Results (Orientation-Dependent Effects of Constant Limbus-Distance Marking)

### 3.1. Constant Distance Assumption in Scleral Fixation Planning

In contemporary scleral fixation techniques, fixation points are frequently marked at a constant distance from the limbus, most commonly 2.5–3.0 mm posteriorly. This strategy implicitly assumes rotational symmetry of the limbal boundary and uniform radial geometry across meridians [11,12,13].

However, the human limbus is characteristically elliptical rather than circular. When the horizontal white-to-white (WTW) diameter exceeds the vertical diameter, the radial distance from a geometrically defined anatomical center to the limbus varies as a function of meridional orientation. Consequently, a constant limbus-based offset does not correspond to a constant circumferential scleral locus.

### 3.2. Analytical Definition of Orientation-Dependent Deviation

Let d(θ) denote the limbus–pars plana distance required to reach a circumferential scleral locus defined relative to a fixed anatomical center.

For an elliptical limbal boundary with semi-axes a (horizontal) and b (vertical), the radial distance from the center to the limbus at meridional angle θ is:

$$r(\theta )=\frac{ab}{\sqrt{(b\cos\theta {)}^{2}+(a\sin\theta {)}^{2}}}$$

If a target scleral locus is defined such that the limbus–pars plana distance equals 3 mm at 0° and 180°, then the effective radial distance to the target locus is:

$$R_{target}=a+3\;mm$$

The orientation-dependent limbus–pars plana distance is therefore:

$$d(\theta )=R_{target}-r(\theta )$$

Deviation from a constant 3 mm marking strategy can then be expressed as:

Δ(θ)=d(θ)−3 mm

By construction, Δ(θ) = 0 at 0° and 180°. When a≠b, Δ(θ) becomes non-zero outside the horizontal meridian and reaches its maximum at 90°.

### 3.3. Magnitude and Deterministic Nature of the Deviation

**Figure 3:**
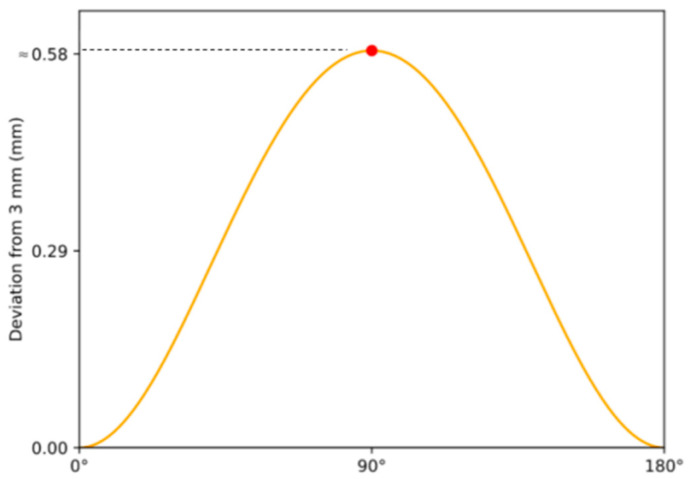
Orientation-dependent deviation from a constant 3 mm limbus-based marking strategy for an eye with a horizontal WTW of 11.8 mm and a vertical WTW of 10.63 mm. The maximum deviation (~0.58 mm) occurs at 90°.

Using representative biometric values (horizontal WTW 11.8 mm; vertical WTW 10.63 mm; thus a = 5.90 mm and b = 5.315 mm), the calculated deviation demonstrates a smooth, symmetric angular dependence. The calculated angular profile of Δ(θ), derived from the elliptical model, is illustrated in Figure 3.

As shown in Figure 3, the deviation is:

- zero at 0° and 180°;
- maximal in the vertical meridian (90°);
- continuous and analytically predictable.

The maximum deviation in this example approaches approximately 0.585 mm, reflecting the difference between the circular and elliptical assumptions.

Importantly, this deviation is not random. It arises deterministically from limbal ellipticity and therefore represents a systematic geometric bias rather than surgical variability [5,6,7].

As a consequence, fixation points placed at identical limbus-based distances across different meridians do not lie on a common circumferential scleral locus. The resulting mismatch is inherent to the geometric model rather than to the surgical technique.

### 3.4. Extension to Patient-Specific Limbal Contours

The limbal boundary is modeled above as an ellipse defined by the horizontal and vertical white-to-white (WTW) diameters. This low-order representation captures the dominant horizontal–vertical anisotropy and allows analytic orientation-dependent planning.

The framework, however, is not restricted to an idealized ellipse. When higher-fidelity anatomical data are available, the limbal contour may be derived from anterior segment imaging modalities such as AS-OCT or Scheimpflug tomography and approximated as a closed spline-interpolated curve [5,14].

**Figure 4:**
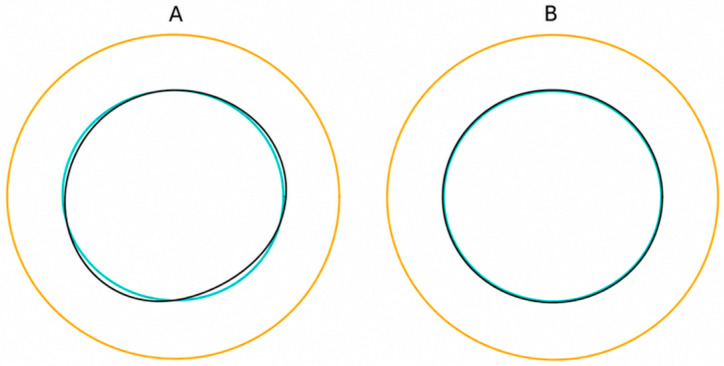
Generalization of the geometric planning framework from an idealized elliptical limbus to patient-specific contour representations. (**A**) Illustrative exaggerated deviation from ellipticity modeled using a smooth spline contour. (**B**) Subtle physiologic deviation representative of realistic biometric variability. In both cases, the same circumferential target locus (orange) is preserved, demonstrating that the planning logic remains unchanged despite contour irregularity.

In this generalized formulation, the anatomical reference center remains unchanged. For each meridian θ, the radial distance to the spline-defined contour is computed from its intersection with the corresponding meridional ray. The target scleral locus is then defined by applying the prescribed limbus-to-pars plana offset along that meridian. Alternatively, a constant normal-offset parallel curve may be constructed to maintain geometric equidistance from the true limbal boundary. The geometric formulation remains valid when the limbal contour is represented by a patient-specific spline model rather than by an ideal ellipse (Figure 4).

Importantly, this substitution does not modify the conceptual structure of the model. Fixation points remain expressible as coordinate pairs (θ, r(θ)) relative to a defined reference center. Thus, the planning framework accommodates both analytic (elliptical) and patient-specific contour-based definitions while preserving geometric consistency across meridians.

## 4. Illustrative Fixation Configurations

### 4.1. Two-Point Fixation

In two-point fixation systems, small meridional asymmetries in effective scleral positioning may translate into IOL tilt or decentration relative to the visual axis. Even submillimeter positional discrepancies have been shown to induce measurable higher-order aberrations, particularly in aspheric intraocular lenses [5,6,7,12].

**Figure 5:**
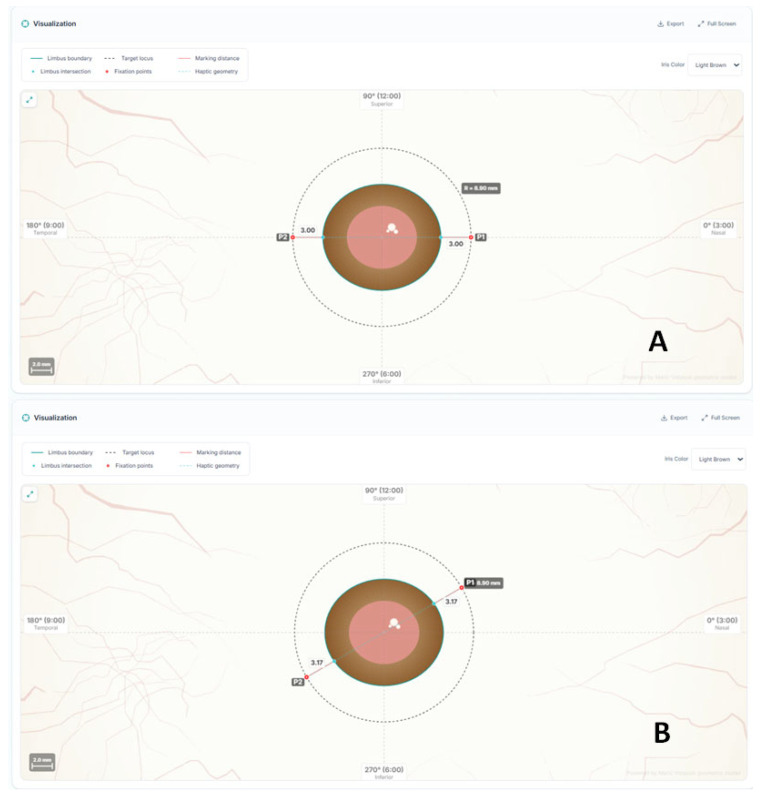
Orientation-dependent planning in a two-point fixation configuration. (**A**) Horizontal fixation at 0°/180°, where a constant 3.0 mm limbus offset corresponds to the intended circumferential scleral locus. (**B**) Rotation of the fixation axis by 30°/210° in an eye with a horizontal WTW of 11.8 mm and a vertical WTW of 10.6 mm. Orientation-adjusted planning increases the required limbus offset to approximately 3.17 mm at both fixation points in order to preserve the same scleral fixation radius. Without this correction, the fixation points would be displaced inward relative to the target geometry and may contribute to a myopic refractive tendency.

When fixation points are placed at 0°/180°, a constant 3.0 mm limbus distance corresponds to a consistent locus. If both points are rotated by 30°, while maintaining a 3.0 mm distance from the limbus, the effective scleral locus shifts inward relative to the intended geometry (Figure 5).

In a two-point configuration, this systematic inward displacement may contribute to anterior displacement of the supported optic plane and a corresponding myopic refractive tendency. The direction of deviation is predictable from Δr(θ).

### 4.2. Three-Point Fixation

In three-point fixation, geometric distortion becomes more complex. Geometric inconsistency across meridians may generate asymmetric tension vectors, potentially affecting lens centration and long-term positional stability. Orientation-dependent deviations at each anchor alter both centration and plane orientation [12,15,16].

**Figure 6:**
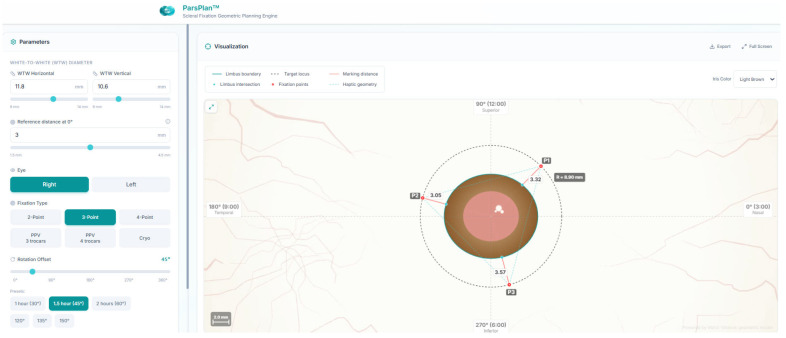
Representative example of orientation-adjusted three-point fixation planning. The limbal boundary was modeled as an ellipse based on horizontal (11.8 mm) and vertical (10.6 mm) white-to-white measurements. For a planned fixation radius of 9.0 mm, three fixation points rotated by 45° required meridian-specific limbus offsets of 3.32 mm, 3.05 mm, and 3.57 mm, respectively. These values differ despite a common reference distance of 3.0 mm because the local limbal radius varies with the meridian. By applying orientation-dependent geometric correction, the software preserves a constant circumferential scleral locus and the geometric symmetry of the fixation construct despite limbal asymmetry.

Because the supporting plane is defined by three points, even small meridional inconsistencies may combine to produce tilt. To illustrate the geometric implications of orientation-dependent deviation, a representative three-point fixation configuration is shown in Figure 6.

In a constant limbus-based marking strategy, fixation points distributed across different meridians do not lie on a common circumferential scleral locus. The resulting geometric inconsistency is expressed as a meridional deviation (Δ), most pronounced outside the horizontal axis.

### 4.3. Four-Point Fixation

In four-point fixation, even small orientation-dependent discrepancies in limbus-based marking propagate across all anchors. Although this configuration enhances rotational stability, circumferential non-uniformity may still displace the fixation complex from a true circular scleral locus, potentially altering the effective lens position and orientation.

Orientation-dependent planning maintains a consistent circumferential locus for all fixation sites [17,18,19,20]. Thus, geometric consistency in scleral fixation is not merely a theoretical construct but may represent a previously under-recognized determinant of optical predictability in secondary IOL implantation.

The examples presented above illustrate that orientation-dependent geometric effects may manifest differently across fixation architectures while arising from the same underlying anatomical asymmetry. A conceptual comparison of representative fixation configurations is provided in Table 1.

## 5. Discussion

### 5.1. Geometric Interpretation of Orientation-Dependent Deviation

The present study formalizes scleral fixation planning as a geometric problem defined by limbal shape and meridional orientation. The central observation is straightforward: a constant limbus-distance strategy assumes rotational invariance of the limbal boundary. In eyes in which the horizontal and vertical WTW differ, that assumption is mathematically inaccurate. The resulting deviation is not stochastic but orientation-dependent and fully predictable from the geometry of the ellipse.

Along the horizontal meridian, circular and elliptical models converge. This explains why fixed-distance marking has appeared clinically acceptable in many traditional horizontal two-point configurations [12]. However, once fixation is rotated toward oblique or vertical meridians—or distributed across multiple meridians in three- or four-point systems—the discrepancy between the circular approximation and elliptical anatomy becomes structurally relevant. The deviation from the intended circumferential locus increases smoothly as a function of meridional orientation and can approach clinically meaningful magnitudes within typical biometric ranges.

The longstanding clinical success of conventional scleral fixation techniques does not invalidate the geometric observation presented here. Multiple sources of biological, optical, and surgical variability may partially mask orientation-dependent effects, particularly in traditional horizontal two-point fixation configurations where the predicted deviation approaches zero. Accordingly, the present framework should not be interpreted as evidence that existing fixation techniques are fundamentally inadequate. Rather, it identifies and formalizes a previously underrecognized source of geometric inconsistency that may contribute to variability in fixation positioning.

The relevance of orientation-dependent planning may become increasingly apparent as secondary IOL fixation strategies evolve beyond conventional horizontal two-point configurations. In three-point, multi-anchor, and particularly emerging artificial capsular support systems, fixation elements are distributed across multiple meridians, including oblique and vertical orientations where deviations arising from a constant limbus-distance strategy become progressively larger. In such architectures, geometric assumptions that may have limited impact in horizontal fixation may become more relevant for maintaining fixation symmetry, implant centration, and fixation-plane stability.

From this perspective, the proposed framework represents not only a correction of a previously underrecognized geometric limitation, but also an analytical foundation for future fixation technologies in which multiple fixation elements are distributed across different meridians around the limbus.

### 5.2. Refractive and Mechanical Implications

From a refractive standpoint, effective lens position (ELP) remains one of the dominant determinants of postoperative outcome [21,22]. Even modest anterior–posterior displacement or subtle tilt can translate into measurable refractive shift, a relationship well established in third-generation IOL power formulas that explicitly predict postoperative lens position. In pseudophakic optics, an anterior displacement of approximately 0.1–0.2 mm may correspond to a refractive change on the order of 0.10–0.25 D, depending on axial length and IOL power [23]. Similar magnitudes of decentration and tilt have been shown to induce higher-order aberrations and reduce optical quality, particularly in aspheric and multifocal IOLs [24], with clinically relevant degradation reported for decentration values exceeding approximately 0.4–0.5 mm and tilt beyond 5–7 degrees [25]. Orientation-dependent deviations approaching 0.5–0.6 mm in representative biometric ranges are within a magnitude that may plausibly influence effective lens position and higher-order aberrations, although this relationship requires prospective clinical validation. Notably, the predicted maximal deviation falls within the magnitudes reported to be associated with clinically detectable optical degradation. In addition to geometric assumptions, manual limbus-based marking using calipers and surgical marking pens may introduce small but non-negligible positional variability, particularly when measurements are transferred across meridians. Such variability may further compound the deterministic orientation-dependent effects described above. Given the well-established sensitivity of refractive outcome to effective lens position, even modest geometric inconsistencies in scleral fixation may translate into clinically meaningful refractive variability. The geometric considerations presented here therefore extend previously described orientation-adjusted fixation strategies into a structured and reproducible analytical framework [15].

Although quantitative effects vary across biometric profiles and lens designs, small positional deviations remain clinically relevant. In multi-point fixation, where deviations may accumulate across meridians, such effects may be amplified. The present model does not suggest that geometric factors are the only source of variability; rather, it isolates one previously under-formalized determinant: meridional mismatch between assumed and actual limbal geometry. By preserving a constant circumferential scleral locus across meridians, the orientation-dependent formulation removes this deterministic geometric component of variability.

### 5.3. Model Characteristics and Theoretical Positioning

The primary objective of this Technical Note is to establish a general analytical framework for orientation-adjusted scleral fixation planning. Clinical validation of its impact on surgical outcomes represents a subsequent stage of investigation. It is important to distinguish between descriptive contour analysis and operative geometric planning. High-order contour descriptions, including Fourier-based representations, are valuable for characterizing limbal or corneal shape irregularities [3,26,27]. However, operative fixation planning requires a spatially intuitive, axis-referenced framework capable of defining discrete anchor points at specific meridional orientations. An elliptical model based on the principal axes provides a low-order, anatomically grounded representation that is directly translatable into surgical coordinates. In this context, the ellipse is not merely an approximation; it is the minimal model capable of capturing the dominant anisotropy of the limbal boundary.

The framework is intentionally technique-independent. The model is not intended to replace or modify IOL power calculation formulas; rather, it defines the scleral fixation coordinates that determine the spatial position of the implanted lens or fixation construct.

Accordingly, the present framework neither calculates the theoretical effective lens position (ELP) used in IOL power calculation formulas nor measures the actual postoperative lens position (ALP). Rather, it defines the geometric fixation coordinates that may influence the eventual postoperative spatial position of the implanted lens. Quantitative assessment of postoperative ALP and its relationship to orientation-adjusted fixation planning will require prospective clinical studies using anterior segment imaging modalities.

Whether fixation is achieved through flanged haptics, sutures, or fixation of a capsular replacement construct, the geometric requirement is identical: anchor points should lie on a common circumferential locus relative to a stable reference center. The model therefore applies equally to two-, three-, and four-point fixation systems and remains valid under circumferential (phase) displacement of fixation points.

### 5.4. Comparison with Existing Personalized Planning Approaches

Several strategies have recently been proposed to improve personalization in scleral-fixated intraocular lens implantation. These approaches generally aim to account for patient-specific ocular dimensions, predicted effective lens position, and the relationship between fixation distance and the overall geometry of the scleral fixation construct.

A recent example is the Personalized Liverpool Scleral-Fixated IOL Calculator (PLiS Fix), which uses a finite-element eye model and biometric parameters to recommend an optimal scleral anchoring point and estimate the resulting horizontal scleral diameter and effective lens position. In the presented model, the fixation distance from the limbus is varied to approximate the horizontal path required for commonly used scleral-fixated IOLs, with predicted horizontal scleral diameter (HSD) and ELP values differing across short, normal, and long eyes [28].

Such approaches represent an important step toward personalized scleral fixation planning because they address inter-eye variability, particularly differences in axial length, global eye size, and predicted postoperative lens position. The geometric framework presented in the present study addresses a distinct and complementary problem: intra-eye orientation-dependent variability arising from horizontal–vertical asymmetry of the limbal boundary.

Rather than estimating the horizontal scleral diameter or directly predicting postoperative ELP, the present model focuses on the geometric definition of the fixation coordinates themselves. Specifically, it evaluates how a fixed limbus-based distance changes its geometric meaning when applied at different meridians in an elliptical limbal model. By explicitly incorporating meridian-dependent limbal geometry, the framework aims to preserve a constant circumferential scleral locus regardless of fixation orientation.

This distinction is clinically relevant because scleral fixation is not limited to a single horizontal axis. Two-point, three-point, and four-point fixation configurations may be rotated or distributed across multiple meridians, particularly in complex secondary IOL implantation and emerging artificial capsular support systems. In such settings, a planning approach based only on global ocular dimensions or horizontal scleral diameter may not fully account for orientation-dependent variation within the same eye.

To our knowledge, no currently published scleral fixation planning framework explicitly incorporates meridian-dependent limbal geometry as a determinant of fixation point calculation.

Recent review articles have identified artificial capsular technologies as an emerging category within secondary intraocular lens fixation strategies. Unlike conventional two-point fixation techniques, these platforms may involve multiple fixation elements whose spatial relationships contribute directly to implant centration and stability. Consequently, geometric planning frameworks capable of accounting for fixation orientation and limbal asymmetry may become increasingly relevant as such technologies evolve and enter broader clinical use [15,29,30].

Therefore, the proposed orientation-adjusted model should be viewed as complementary rather than competitive with existing personalized planning strategies. Future digital surgical planning environments may combine biometric optimization, effective lens position prediction, IOL-specific dimensional modeling, and meridian-specific geometric correction within a unified workflow.

### 5.5. Clinical Translation and Practical Application

The geometric effect described in the present study can be translated into clinical practice through both conventional surgical marking techniques and future image-guided planning systems. The proposed framework does not alter the principles of scleral fixation itself; rather, it provides a method for calculating fixation coordinates that account for orientation-dependent limbal geometry and preserve a constant circumferential scleral locus across different meridians.

In conventional surgery, fixation points are typically determined using calipers and limbus-based manual marking techniques. Although these methods remain practical and widely adopted, the transfer of geometric measurements to the ocular surface is inherently subject to operator-dependent variability [31]. Sources of imprecision may include caliper positioning, marker width, ink diffusion on the ocular surface, partial obscuration of marks during surgery, and geometric inaccuracies introduced when measurements are transferred across different meridians. Such variability becomes increasingly relevant when submillimeter positional differences are considered [32]. Furthermore, the geometric meaning of a fixed limbus-based distance changes according to the meridional orientation in eyes with elliptical limbal anatomy, potentially introducing systematic deviations from the intended fixation geometry.

The framework may be applied to a broad range of scleral fixation techniques, including two-point fixation methods such as Yamane-type fixation, sutureless intrascleral haptic fixation, and flange-based techniques, as well as four-point fixation approaches employing sutured fixation systems. Potential future applications may also include emerging three-point fixation concepts and artificial capsular support systems, where geometric symmetry among fixation points may become increasingly important for maintaining centration and minimizing tilt.

From a practical perspective, the required correction can be calculated preoperatively using horizontal and vertical white-to-white measurements and subsequently translated into intraoperative fixation coordinates. While simplified correction tables and caliper-based approaches may facilitate adoption in conventional surgical settings, digital implementation offers a more direct and reproducible pathway for applying orientation-adjusted planning in clinical practice.

A particularly attractive application lies within image-guided ophthalmic surgical platforms. Similar approaches have already been successfully adopted for toric intraocular lens alignment, where digital registration and intraoperative overlay systems have largely complemented conventional manual marking by improving reproducibility and reducing alignment variability [31,33]. In principle, the same workflow may be extended to scleral fixation planning.

Because the fixation coordinates generated by the present framework are defined mathematically as meridian-specific spatial coordinates relative to a reference center, they are inherently compatible with digital registration, eye-tracking, and intraoperative overlay technologies [34]. Following acquisition of horizontal and vertical white-to-white measurements, fixation coordinates could be calculated automatically and projected directly onto the surgical field through microscope-integrated image-guidance systems. Such an approach would eliminate the need for manual transfer of meridian-specific corrections and would allow continuous compensation for ocular rotation, cyclotorsion, and changes in eye position during surgery [33,35].

Importantly, the proposed framework should be viewed as a geometric planning aid rather than a replacement for surgical judgment or technique-specific decision-making. The final position, centration, and stability of a scleral-fixated intraocular lens remain influenced by multiple intraoperative and postoperative factors that extend beyond fixation point geometry alone.

### 5.6. Digital Implementation (ParsPlan)

To facilitate the practical application of the proposed framework, the analytical model was implemented as a software prototype (ParsPlan). Based on horizontal and vertical white-to-white measurements, the platform reconstructs an individualized limbal ellipse and calculates orientation-adjusted fixation coordinates for two-point, three-point, and four-point fixation configurations. Meridian-specific limbus offsets are determined automatically in order to preserve a constant circumferential scleral locus despite limbal asymmetry. The current prototype performs deterministic calculations based on user-entered biometric parameters and displays the resulting fixation coordinates numerically and graphically for preoperative planning. The software serves as a proof-of-concept implementation of the analytical framework presented in this study, allowing interactive visualization of orientation-dependent fixation planning under different biometric conditions. At its present stage, the application is intended as a research and planning tool rather than a clinically validated surgical decision-support system. Its primary purpose is to demonstrate the feasibility of translating the proposed analytical framework into a practical computational workflow. Future developments may include implementation as an online personalized planning platform and integration with image-guided surgical navigation systems capable of displaying patient-specific fixation coordinates intraoperatively.

**Figure 7:**
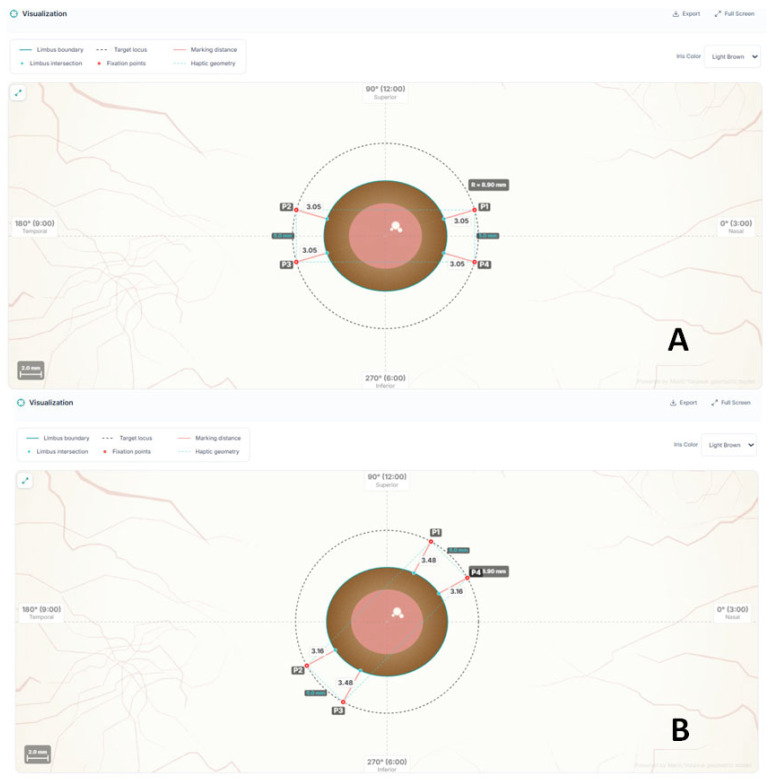
Comparison of conventional (**A**) and orientation-adjusted (**B**) planning in a four-point fixation configuration. Baseline horizontal distances are 3 mm from the limbus and 5 mm between ipsilateral haptic positions (e.g., P2 and P3). Uniform limbus-distance marking produces meridian-dependent variation in effective scleral positioning and loss of geometric symmetry of the fixation construct. Orientation-adjusted planning compensates for limbal ellipticity and maintains all fixation points on a common circumferential scleral locus.

Representative examples generated using the software prototype are presented in Figure 5, Figure 6 and Figure 7.

## 6. Limitations and Future Directions

### 6.1. Limitations

Several limitations merit acknowledgment. The present analysis is theoretical and does not incorporate biomechanical factors such as scleral elasticity, suture tension, or postoperative remodeling. The model also assumes accurate measurement of the horizontal and vertical WTW diameters and a stable anatomical reference center.

#### Sensitivity to WTW Measurement Error

The maximum orientation-dependent deviation occurs at the vertical meridian and reduces to a simple difference term:

Δmax=(WTW horizontal−WTW vertical)/2.

Accordingly, any measurement uncertainty in the WTW propagates directly into the predicted correction. For example, if each WTW diameter is uncertain by ±0.2 mm, then Δmax carries up to ±0.2 mm uncertainty in the worst case (opposing errors). Published repeatability and inter-device agreement for WTW measurements vary, with inter-device limits of agreement approaching ~0.5 mm in some comparisons [36,37].

For practical implementation, the additional posterior offset required at 90° relative to a standard limbus-based mark approximates Δmax under typical biometric conditions (Table 2).

Although the proposed framework has been translated into a functional computational workflow, its clinical value remains to be established through prospective clinical validation. Preliminary clinical experience with geometry-informed fixation planning has been reported as a feasibility demonstration in small case series rather than as formal validation [15]. The present study demonstrates geometric inconsistency rather than clinical superiority. Whether correction of this inconsistency translates into measurable improvements in postoperative refractive outcomes remains to be established prospectively.

### 6.2. Future Directions

Future studies should compare orientation-adjusted and conventional fixation planning by correlating predicted fixation coordinates with postoperative measurements of IOL centration, tilt, and refractive outcomes obtained using anterior segment OCT or Scheimpflug imaging [38,39]. Such investigations will determine the extent to which meridian-dependent geometric correction contributes to improved positional stability and refractive predictability.

Nevertheless, the geometric principle underlying the proposed framework remains independent of the magnitude of the clinical outcome. When the limbus is elliptical, a constant limbus-distance rule cannot preserve a uniform circumferential scleral locus across meridians. Orientation-adjusted planning therefore provides an anatomy-based method for incorporating meridian-dependent limbal geometry into fixation planning.

## 7. Conclusions

Modeling the limbus as elliptical rather than circular reveals an inherent orientation-dependent limitation of constant limbus-distance planning. An explicit geometric formulation based on r(θ) and Δr(θ) preserves a constant circumferential scleral locus across meridians and across fixation configurations.

The proposed framework can be translated into a practical computational workflow suitable for individualized surgical planning and future image-guided applications. This technique-independent framework provides a mathematically transparent foundation for scleral fixation planning within a pars plana surgical context.

Prospective clinical validation will be required to determine the impact of orientation-adjusted planning on postoperative lens centration, tilt, and refractive predictability.

## 8. Patents

Goran Marić and Zoran Vatavuk are the owners of patent application No. HR P20260227A.

## Figures and Tables

**Table 1:** Geometric implications of orientation-dependent fixation planning in representative scleral fixation configurations. The relative sensitivity to meridional limbal asymmetry increases with the complexity and spatial distribution of fixation points, potentially influencing implant centration, plane orientation, and overall fixation symmetry.

Fixation Pattern	Number of Points	Orientation Sensitivity	Potential Consequence
2-point **	2	Moderate	Tilt, Myopic shift
3-point ***	3	High	Plane tilt, Asymmetric tension
4-point ****	4	Moderate—High	Rotational asymmetry, Geometric distortion

** Examples include Yamane and Carlevale IOL fixation techniques. *** Examples include artificial capsular support platforms and other multi-anchor fixation systems. **** Examples include sutured four-point fixation techniques (e.g., Gore-Tex fixation).

**Table 2:** Simple correction at the vertical meridian based on the horizontal–vertical WTW difference (Δmax).

WTWh—WTWv (mm)	Add to Standard Limbus-Based Offset at 90° (mm)
0.5	0.25
1.0	0.50
1.5	0.75
2.0	1.00

## Data Availability

The raw data supporting the conclusions of this article will be made available by the authors on request.

## Abbreviations

The following abbreviations are used in this manuscript:

WTW	White-To-White
IOL	Intraocular Lens
ELP	Effective Lens Position
ALP	Actual Postoperative Lens Position
AS-OCT	Anterior Segment Optical Coherence Tomography
IT	Information Technology
HSD	Horizontal Scleral Diameter
